# Evidence for Altered Metabolism of Sphingosine-1-Phosphate in the Corpus Callosum of Patients with Schizophrenia

**DOI:** 10.1093/schbul/sbaa052

**Published:** 2020-04-29

**Authors:** Kayoko Esaki, Shabeesh Balan, Yoshimi Iwayama, Chie Shimamoto-Mitsuyama, Yoshio Hirabayashi, Brian Dean, Takeo Yoshikawa

**Affiliations:** 1 Laboratory for Molecular Psychiatry, RIKEN Center for Brain Science, Saitama, Japan; 2 Support Unit for Bio-Material Analysis, Research Division, RIKEN Center for Brain Science, Saitama, Japan; 3 Cellular Informatics Laboratory, RIKEN Cluster for Pioneering Research, Saitama, Japan; 4 The Florey Institute of Neuroscience and Mental Health, Howard Florey Laboratories, The University of Melbourne, Victoria, Australia; 5 The Centre for Mental Health, Swinburne University, Victoria, Australia

**Keywords:** schizophrenia, corpus callosum, sphingosine-1-phosphate (S1P), S1P receptor, gene expression

## Abstract

The disturbed integrity of myelin and white matter, along with dysregulation of the lipid metabolism, may be involved in schizophrenia pathophysiology. Considering the crucial role of sphingolipids in neurodevelopment, particularly in oligodendrocyte differentiation and myelination, we examined the role of sphingolipid dynamics in the pathophysiology of schizophrenia. We performed targeted mass spectrometry-based analysis of sphingolipids from the cortical area and corpus callosum of postmortem brain samples from patients with schizophrenia and controls. We observed lower sphingosine-1-phosphate (S1P) levels, specifically in the corpus callosum of patients with schizophrenia, but not in major depressive disorder or bipolar disorder, when compared with the controls. Patient data and animal studies showed that antipsychotic intake did not contribute to the lowered S1P levels. We also found that lowered S1P levels in the corpus callosum of patients with schizophrenia may stem from the upregulation of genes for S1P-degrading enzymes; higher expression of genes for S1P receptors suggested a potential compensatory mechanism for the lowered S1P levels. A higher ratio of the sum of sphingosine and ceramide to S1P, which can induce apoptosis and cell-cycle arrest, was also observed in the samples of patients with schizophrenia than in controls. These results suggest that an altered S1P metabolism may underlie the deficits in oligodendrocyte differentiation and myelin formation, leading to the structural and molecular abnormalities of white matter reported in schizophrenia. Our findings may pave the way toward a novel therapeutic strategy.

## Introduction

Although the neurobiological mechanism of schizophrenia remains largely elusive, reduced white matter in the brain has been consistently reported in patients with schizophrenia and shown to be correlated with negative symptoms.^1–4^ In particular, diffusion tensor imaging (DTI), which measures water diffusion within the axon or myelin sheath, showed a significant reduction in fractional anisotropy (FA) in regions of the brain, including the corpus callosum, in patients with schizophrenia.^4–7^ This decrease in FA may indicate abnormalities of myelination and oligodendrocyte functions; postmortem brain tissues of patients with schizophrenia revealed impairments of the myelin-sheath lamellae, reduction in the area of the nucleus and the volume density of mitochondria in oligodendrocytes, and downregulation of myelin-related genes.^8–10^ However, the mechanism underlying these observations is unclear. Recently, several studies have reported an altered metabolism of lipids^11,12^ and amino acid serine^13,14^ in postmortem brain and blood samples, respectively, from patients with schizophrenia. Of note, metabolic abnormalities of sphingolipids, which are serine-derived lipids, were also observed.^15–17^

**Fig. 1.**
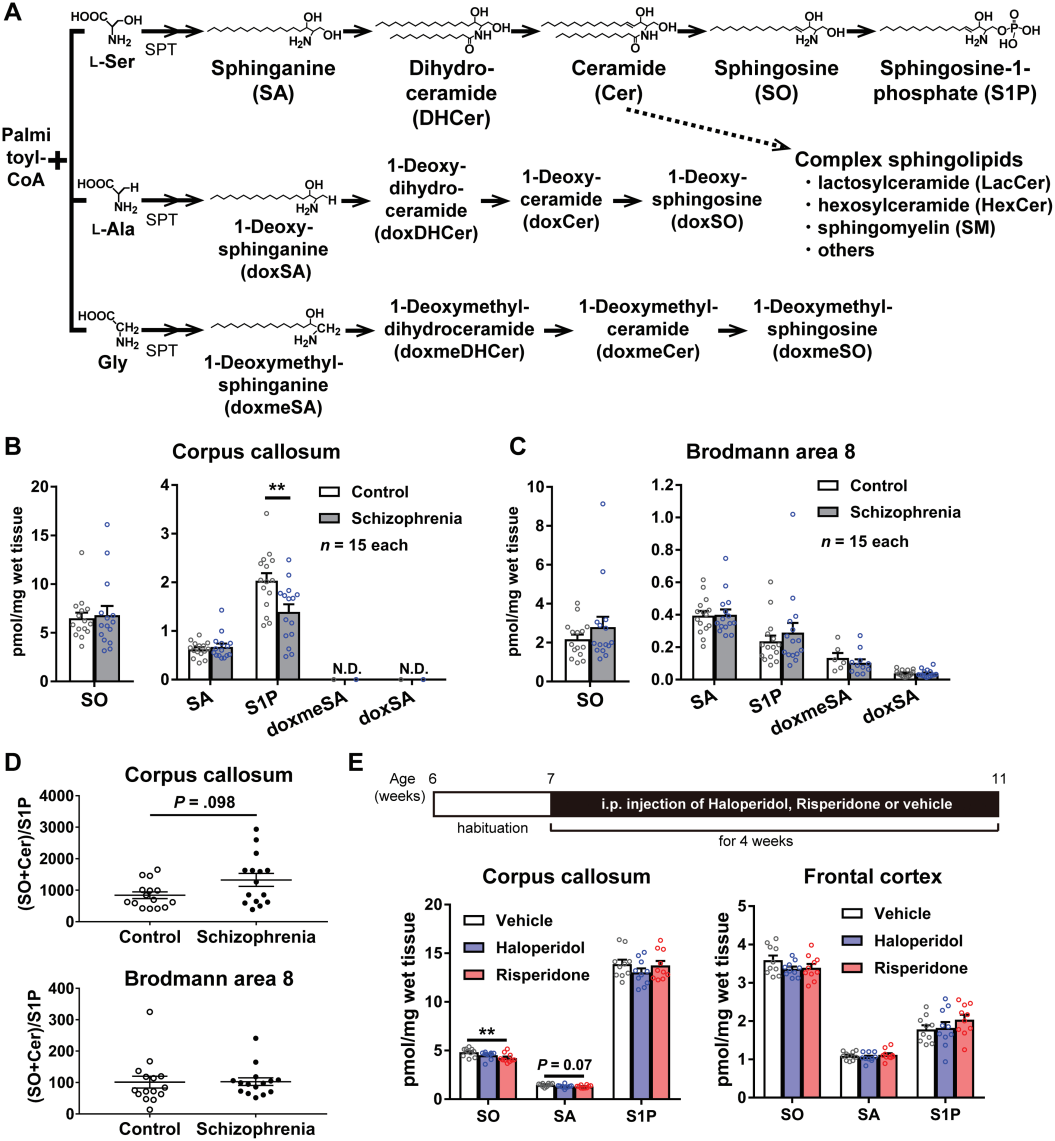
Sphingoid base levels in postmortem human and mouse brains. (A) Sphingolipid synthesis pathway. Sphingolipid biosynthesis starts with the condensation of palmitoyl-CoA and l-serine by serine palmitoyltransferase (SPT). Then, sphinganine (SA), dihydroceramide (DHCer), ceramide (Cer), sphingosine (SO), and sphingosine-1-phosphate (S1P) are serially synthesized. Furthermore, not only l-serine (Ser) but also l-alanine (Ala) or glycine (Gly) is used to generate sphingoid bases. Lipids were analyzed for sphingoid base levels from (B) the corpus callosum and (C) Brodmann area 8. S1P levels are low in the corpus callosum of patients with schizophrenia. The levels of doxSO and doxmeSO are below the detection limits in the corpus callosum and BA8, limiting their quantification. (D) The ratio of the sum of SO and Cer to S1P in the human corpus callosum and Brodmann area 8. (B–D) Data are represented as the mean ± SEM. N.D., not determined; **P < .01. Differences between 2 groups were analyzed by Mann-Whitney *U* test. (E) Administration of antipsychotic drugs did not affect S1P levels in the corpus callosum and frontal cortex of mice. Data are represented as the mean ± SEM. **P < .01. Differences among 3 groups were analyzed by non-parametric Kruskal-Wallis analysis, followed by Dunnett’s test (vs vehicle group).

Sphingolipids form the key structural component of plasma membranes and are essential for the development and normal function of the brain. Biosynthesis of most sphingolipids starts with the condensation of palmitoyl-CoA and amino acid l-serine by serine palmitoyltransferase (SPT), and some with alanine or glycine (the final product is 1-deoxy-sphinganine or 1-deoxymethyl-sphinganine) (figure 1A).^18–22^ There are 2 types of sphingolipids: base form and fatty-acid-acylated form. The base form includes sphingosine-1-phosphate (S1P) and sphingosine (SO), and the fatty-acid-acylated form includes ceramide (Cer) and sphingomyelin (SM) (figure 1A). Sphingosine-1-phosphate is serine-derived and acts as a signaling molecule; it is involved in the regulation of various functions, such as myelination, differentiation of oligodendrocytes, hematopoietic cell trafficking, autophagy, and immune-cell fate via S1P receptors.^23–25^

Previous studies have shown that levels of SM and Cer were reduced in postmortem brain and skin samples, respectively, from patients with schizophrenia.^16,26^ Furthermore, an altered expression level of the gene for SPT long chain base subunit 2 (*SPTLC2*), 1 of 3 different subunits of SPT, was observed in brain samples from patients with schizophrenia.^27^ However, the role of sphingolipids in schizophrenia has not been sufficiently addressed.

We performed a systematic analysis of sphingolipid levels and an expression analysis of genes for sphingolipid metabolism in the corpus callosum (representative white matter) and Brodmann area 8 (BA8; frontal cortical area) of postmortem brain samples from patients with schizophrenia and age-/sex-matched controls. Subsequently, we looked for altered sphingolipids in postmortem brain samples from patients with major depression and bipolar disorder and compared them with the controls to determine whether the dysregulation is specific to schizophrenia or a global phenomenon seen in other psychiatric disorders. Furthermore, we used mice to examine the effects of antipsychotics on the sphingolipid metabolism.

## Materials and Methods

### Postmortem Brain Samples

Samples of the corpus callosum and BA8 were obtained from the Victorian Brain Bank Network at the Florey Institute for Neuroscience and Mental Health.^28–30^ All procedures were carried out with written informed consent from the next of kin, and the study was approved by the Ethics Committee of RIKEN. After reviewing the clinical records, psychiatric diagnoses were confirmed based on the Diagnostic and Statistical Manual of Mental Disorders (DSM-IV) criteria and the Diagnostic Instrument for Brain Studies, which allow for a consensus psychiatric diagnosis to be made after death.^31,32^ The case histories for all nonpsychiatric subjects (controls) were extensively reviewed, and neuropsychopharmacological profiles were obtained. Treating clinicians and family members were also interviewed to exclude any history of psychiatric illness in the controls.

We first quantified sphingolipids in the postmortem brain samples from patients with schizophrenia and the sphingolipids with altered levels were subsequently evaluated in the patients with major depression and bipolar disorder, in comparison to the controls (supplementary figure S1). For sphingolipid analysis, we used frozen tissue samples from patients with (1) schizophrenia (*n* = 15, supplementary table S1), (2) major depressive disorder (*n* = 15, supplementary table S2), (3) bipolar disorder (*n* = 15, supplementary table S3), and (4) non-affected controls (*n* = 15, supplementary table S4) (sample set 1, table 1). Within each control-schizophrenia-major depressive disorder-bipolar disorder tetrad (total of 15 tetrads), the age at death, sex, and postmortem interval were strictly matched; we also ensured there were no significant differences in those measures between the test groups and the controls (table 1).^33^

**Table 1.** Characteristics of Postmortem Brain Sample Set 1 For Lipid Analysis

	Control (*n* = 15)	Schizophrenia (*n* =15)	Depression (*n* =15)	Bipolar Disorder (*n* =15)
Age at death (y)	57.3 ± 13.0	57.9 ± 13.7	57.4 ± 12.3	58.5 ± 13.6
Sex (male/female)	8/7	8/7	8/7	8/7
Duration of illness (y)		24.6 ± 14.3	17.8 ± 9.5	16.7 ± 11.5
Postmortem interval (h)	43.1 ± 17.7	42.9 ± 12.8	42.1 ± 16.8	36.4 ± 15.3
Brain tissue pH	6.34 ± 0.23	6.18 ± 0.27	6.52 ± 0.19	6.26 ± 0.24

*Note*: The values are represented as the mean ± SD. Differences among 4 groups were analyzed using the non-parametric Kruskal-Wallis 1-way analysis of variance, followed by Dunnett’s test (vs control group). There were no significant differences.

For gene expression analysis, we used an expanded sample set from the corpus callosum and BA8 of patients with schizophrenia (*n* = 91) and controls (*n* = 90). The expanded sample set also included sample set 1 (sample set 2, supplementary table S5).^33^ The overall flow of the experiment is shown in supplementary figure S1.

### Animals

All animal experiments were performed in compliance with relevant laws and the guidelines approved by the Animal Ethics Committee at RIKEN. Male C57BL/6J mice (6 weeks old) were intraperitoneally administered phosphate-buffered saline (vehicle), haloperidol (0.1 mg/kg body weight), or risperidone (0.2 mg/kg body weight) for 4 weeks. After the mice were euthanized by cervical dislocation, the brains (vehicle, *n* = 10; haloperidol, *n* = 10; risperidone, *n* = 10) were excised as quickly as possible and stored at −80°C until the frontal cortex and corpus callosum were dissected for the downstream analyses. Methodology details are provided as supplementary information.

### Measurement of Sphingolipids

Sphingolipids were extracted from the brain tissues of both humans and mice (Bligh and Dyer method)^34^ and quantified using liquid chromatography-electrospray ionization-tandem mass spectrometry (LC-ESI-MS/MS).^18^Methodology details are provided as supplementary information.

### Gene Expression Analysis

Expression of the target genes was measured by quantitative real-time reverse-transcription polymerase chain reaction (RT-PCR) or digital PCR using TaqMan Gene Expression Assays as described elsewhere.^35,36^ Expression of the selected target genes in the corpus callosum were quantified by normalizing with the geometric mean of Glyceraldehyde-3-Phosphate Dehydrogenase (*GAPDH*) and Beta-2-Microglobulin (*B2M*) expression in the final evaluation. The overall flow of gene expression analysis is shown in supplementary figure S2.

Absolute quantification of mRNAs was performed in the corpus callosum and BA8/frontal cortex of samples from human controls (*n* = 6) and C57BL/6J mice (*n* = 3), using digital PCR. Methodology details are provided as supplementary information.

### Statistics

GraphPad Prism version 7 (GraphPad Software) was used for data analysis. Data are presented as the mean ± standard error of the mean (SEM) or as the mean ± standard deviation (SD). Outliers (more or less than the mean ± 2SD) were excluded. Significant changes between 2 groups were tested using the Mann-Whitney *U* test (2-tailed). Differences among more than 2 groups were analyzed using the nonparametric Kruskal–Wallis H test, followed by Dunnett’s test for all data, including mouse lipid data, which did not show normality with the D′Agostino-Pearson test. Correlation was examined using Spearman’s rank correlation coefficient. *P* < .05 was considered statistically significant and .05 ≤ *P* < .1 was considered a trend toward significance.

## Results

### Levels of Sphingosine-1-Phosphate Were Lowered in the Corpus Callosum of Patients With Schizophrenia

The study design is shown in supplementary figure S1. We first analyzed sphingolipids in the corpus callosum (white matter) and BA8 (cortical area) of postmortem brain samples from patients with schizophrenia and controls using the LC-ESI-MS/MS method.^18^ We detected significantly lower levels of S1P, 1 of the base-form species of sphingolipids, in the corpus callosum of subject with schizophrenia than in the controls (31% reduction, *P* = .009) (figure 1B). By contrast, the levels of other base-form species of sphingolipids (sphinganine [SA], SO, 1-deoxy-sphinganine, and 1-deoxymethyl-sphinganine) did not differ between the schizophrenia and control groups (figures 1B and 1C). We also analyzed fatty-acid-acylated forms of sphingolipids (dihydroceramide, Cer, 1-deoxy-dihydroceramide, 1-deoxy-ceramide, 1-deoxymethyl-dihydroceramide, 1-deoxymethyl-ceramide [doxmeCer], lactosylceramide, hexosylceramide, and SM) (figure 1A). In the corpus callosum, none of the acylated forms of sphingolipids differed between the schizophrenia and control groups (table 2 and supplementary table S6). Although higher levels of Cer and doxmeCer, which contain fatty acids of various chain lengths, were observed in the BA8 of patients with schizophrenia than in that of the controls, the levels of the other fatty-acid-acylated species were unchanged (table 2 and supplementary table S7).

**Table 2.** Levels of Sphingolipids in the Corpus Callosum and Brodmann Area 8 From the Subjects With Schizophrenia and Controls

*N*-Acyl chain		C14	C16	C18	C18:1	C20	C20:1	C22	C22:1	C24	C24:1	C26	C26:1	SUM
Corpus callosum														
Cer	CON	0.34 ± 0.04	6.54 ± 0.46	276.80 ± 20.71	0.65 ± 0.07	26.70 ± 2.56	1.26 ± 0.12	63.56 ± 6.48	16.32 ± 1.78	130.34 ± 19.87	984.43 ± 118.38	7.15 ± 0.96	56.75 ± 10.03	1570.83 ± 172.66
	SCZ	0.41 ± 0.05	6.08 ± 0.55	297.64 ± 29.55	0.66 ± 0.06	26.95 ± 3.48	1.27 ± 0.10	68.00 ± 9.54	17.21 ± 2.25	150.82 ± 26.61	903.77 ± 145.44	6.94 ± 1.08	65.83 ± 11.35	1545.54 ± 215.00
	*P*	0.38	0.44	0.54	0.87	0.74	0.97	>0.99	0.97	0.57	0.39	0.65	0.57	0.65
SM	CON	0.01 ± 0.00	0.42 ± 0.03	2.79 ± 0.21	0.25 ± 0.02	0.24 ± 0.02	0.02 ± 0.00	0.21 ± 0.02	0.11 ± 0.01	0.82 ± 0.09	3.73 ± 0.31	0.07 ± 0.01	0.57 ± 0.06	9.23 ± 0.71
	SCZ	0.01 ± 0.00	0.39 ± 0.03	2.56 ± 0.18	0.22 ± 0.01	0.22 ± 0.02	0.02 ± 0.00	0.21 ± 0.02	0.10 ± 0.01	0.74 ± 0.06	3.74 ± 0.34	0.06 ± 0.01	0.53 ± 0.05	8.80 ± 0.66
	*P*	0.90	0.39	0.39	0.16	0.29	0.81	>0.99	0.37	0.49	0.97	>0.99	0.57	0.57
DoxmeCer	CON	N.D.	0.51 ± 0.05	11.92 ± 1.08	0.17 ± 0.02	0.20 ± 0.02	N.D.	0.74 ± 0.08	N.D.	10.39 ± 1.04	3.66 ± 0.30	1.81 ± 0.21	4.36 ± 0.31	33.67 ± 2.10
	SCZ	N.D.	0.49 ± 0.04	11.55 ± 0.96	0.15 ± 0.02	0.20 ± 0.02	N.D.	0.81 ± 0.09	N.D.	9.96 ± 1.38	3.98 ± 0.63	1.52 ± 0.18	4.21 ± 0.44	32.79 ± 2.74
	*P*		0.87	0.90	0.23	0.64		0.30		0.68	>0.99	0.34	0.93	0.74
Brodmann area 8														
Cer	CON	0.08 ± 0.01	0.32 ± 0.05	11.06 ± 1.29	0.02 ± 0.00	0.62 ± 0.07	1.90 ± 0.33	0.19 ± 0.03	0.00 ± 0.00	0.20 ± 0.07	1.08 ± 0.26	0.01 ± 0.00	0.13 ± 0.04	15.62 ± 1.73
	SCZ	0.11 ± 0.01	0.50 ± 0.06	14.26 ± 1.19	0.04 ± 0.01	0.80 ± 0.10	3.16 ± 0.60	0.29 ± 0.05	0.01 ± 0.00	0.31 ± 0.13	2.10 ± 0.82	0.01 ± 0.01	0.19 ± 0.08	21.79 ± 2.62
	*P*	0.002	0.008	0.02	0.02	0.22	0.13	0.09	0.48	0.30	0.25	0.46	0.29	0.07
SM	CON	0.01 ± 0.00	0.38 ± 0.04	4.30± 0.58	0.50 ± 0.06	2.57 ± 0.40	0.10 ± 0.02	0.29 ± 0.04	0.10 ± 0.02	0.59 ± 0.09	3.64 ± 0.60	0.05 ± 0.01	0.54 ± 0.09	13.07 ± 1.65
	SCZ	0.01 ± 0.00	0.43 ± 0.05	4.86 ± 0.69	0.55 ± 0.08	2.77 ± 0.39	0.11 ± 0.02	0.32 ± 0.04	0.11 ± 0.02	0.56 ± 0.11	3.18 ± 0.71	0.04 ± 0.01	0.43 ± 0.08	13.37 ± 1.85
	*P*	0.13	0.46	0.51	0.74	0.49	0.44	0.51	0.97	0.54	0.44	0.20	0.39	0.93
Doxme Cer	CON	N.D.	0.27 ± 0.09	2.51 ± 0.54	0.20 ± 0.03	0.17 ± 0.09	1.00 ± 0.21	0.16 ± 0.03	N.D.	0.51 ± 0.12	0.60 ± 0.11	0.26 ± 0.06	0.50 ± 0.13	4.37 ± 0.65
	SCZ	N.D.	0.49 ± 0.17	2.95 ± 0.54	0.16 ± 0.04	0.14 ± 0.05	0.93 ± 0.14	0.33 ± 0.06	N.D.	0.78 ± 0.18	1.99 ± 0.76	0.54 ± 0.40	0.48 ± 0.09	7.01 ± 0.94
	*P*		0.20	0.43	0.46	0.80	0.95	0.01		0.19	0.04	0.63	0.92	0.04

*Note*: The values are represented as the mean ± SEM (*n* = 15 each). The concentrations are shown as pmol/mg wet tissue (ceramide [Cer]), nmol/mg wet tissue (sphingomyelin [SM]), or fmol/mg wet tissue (1-deoxymethyl-dihydroceramide [doxmeCer]). Differences between 2 groups were analyzed by Mann-Whitney *U* test. CON, control; SCZ, schizophrenia; N.D., not detected.

Based on the results of lowered levels of S1P in the corpus callosum of patients with schizophrenia when compared with the controls, we examined S1P levels in the samples of major depressive disorder and bipolar disorder. However, we detected no significant differences of S1P levels in the corpus callosum and BA8 between patients with major depressive disorder or bipolar disorder and controls (supplementary figure S3), indicating that the dysregulation of S1P level is specific to the corpus callosum of patients with schizophrenia.

An elevation in the ratio of the sum of SO and Cer to S1P induces apoptosis, cell-cycle arrest, and the suppression of cell survival and cell proliferation.^37^ We found the ratio displayed a higher trend in the corpus callosum from patients with schizophrenia than in that of controls (figure 1D).

### Lowered S1P Levels in the Corpus Callosum Were not Influenced by Antipsychotic Intake

Since altered levels of S1P, Cer, and doxmeCer were observed in the brain samples of patients with schizophrenia, we further tested the correlation between each potential confounding factor (pH, age, postmortem interval, drug dose, illness duration) and S1P levels in the schizophrenia samples and the pooled samples (control + schizophrenia). We did not find any significant correlations between the confounding factors and the levels of Cer or doxmeCer in the BA8 (supplementary figures S4A and S4B). There was no significant correlation (*P* = .054) between drug dose (chlorpromazine equivalents) and S1P levels in the schizophrenia group (*n* = 15). According to the clinical information, 4 of the patients with schizophrenia were not on medication (supplementary table S1). When patients with schizophrenia (*n* = 11), excluding those not taking antipsychotics, were analyzed, a positive correlation between S1P level and drug dose was detected (*P* = .004) (supplementary figure S4C). However, we cannot rule out a history of antipsychotic intake during the course of disease. Collectively, antipsychotic intake is unlikely to explain the lowered levels of S1P in the patients with schizophrenia.

To further evaluate the effects of antipsychotic drugs on S1P content in the brain, we analyzed sphingolipid levels in the frontal cortex and corpus callosum of mice administered haloperidol or risperidone for 4 weeks. There were no changes in the levels of S1P in the corpus callosum and frontal cortex upon administration of haloperidol or risperidone (figure 1E). The injection of risperidone elicited a significant reduction in SO and a trend of decreasing SA content in the corpus callosum, whereas SO and SA levels were unchanged in the frontal cortex (figure 1E). Thus, lowered S1P levels in the corpus callosum of patients with schizophrenia are unlikely to result from drug administration, though we cannot completely exclude the possibility of antipsychotic drug effects, including those of long-term exposure, on the alteration of S1P content in patients with schizophrenia.

### Expression of Genes for S1P-Degrading Enzymes Is Upregulated in the Corpus Callosum of Patients With Schizophrenia

**Fig. 2.**
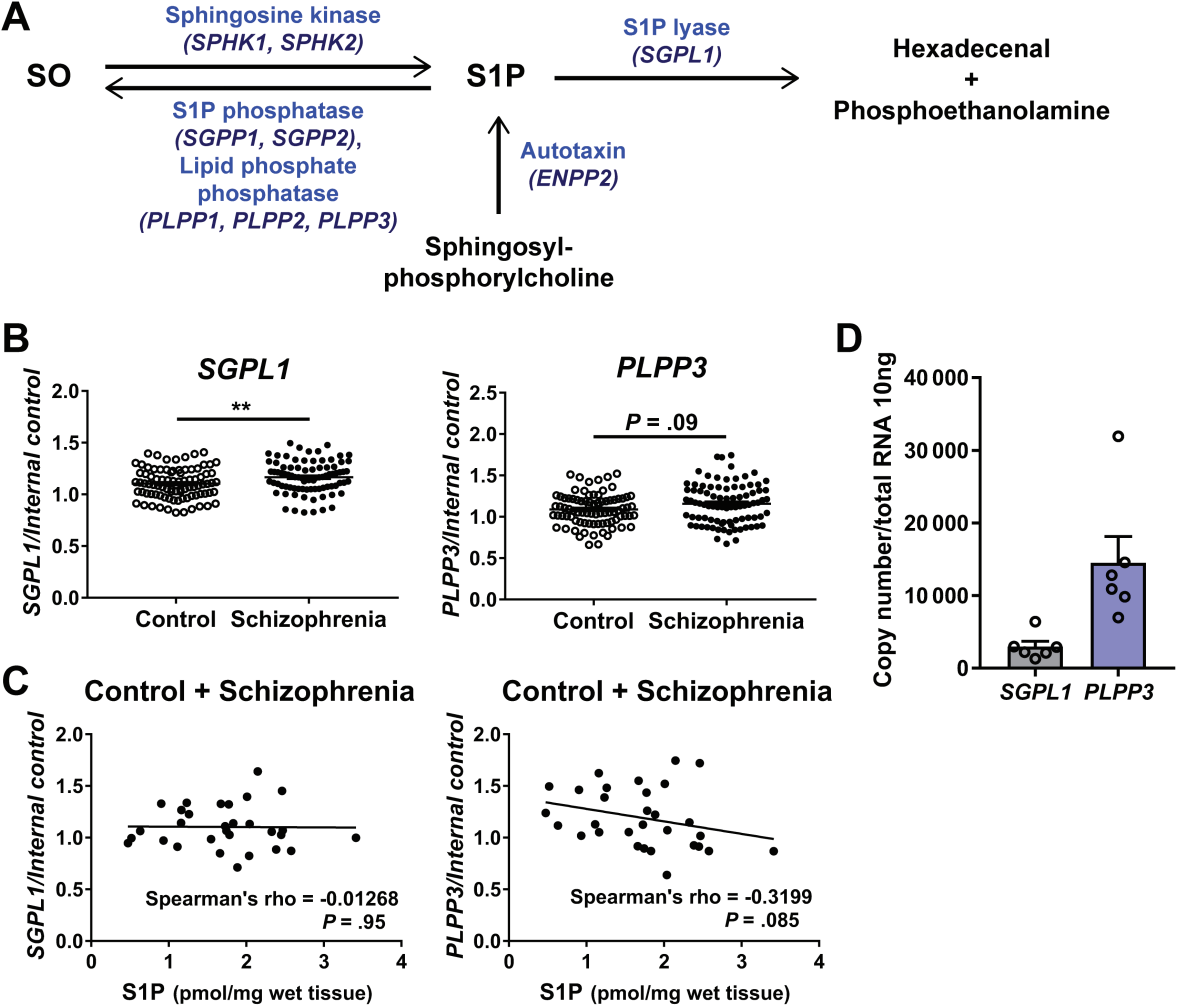
Expression analysis of sphingolipid metabolism-related genes in postmortem human corpus callosum. (A) Metabolic pathway of sphingosine-1-phosphate (S1P). S1P is synthesized from sphingosine or sphingosylphosphorylcholine by sphingosine kinase (encoded by *SPHK1* and *SPHK2*)^38^ or autotaxin (*ENPP2*),^39^ respectively, and degraded to sphingosine or hexadecenal and phosphoethanolamine by S1P phosphatase (*SGPP1* and *SGPP2*) and lipid phosphate phosphatase (*PLPP1*, *PLPP2*, and *PLPP3*) or S1P lyase (*SGPL1*), respectively.^38^ (B) Transcript expression levels of the genes for S1P-metabolizing enzymes in the corpus callosum of patients with schizophrenia (*n* = 91) and controls (*n* = 90). Data were normalized with the geometric mean of the 2 internal control genes (*GAPDH* and *B2M*) and are represented as the mean ± SEM. **P < .01. Differences between 2 groups were analyzed by Mann-Whitney *U* test. (C) Correlations between S1P levels and transcript expression levels of *SGPL1* and *PLPP3* in the corpus callosum of patients with schizophrenia and controls (Spearman’s rank correlation coefficient). (D) Absolute quantification of expression levels of *SGPL1* and *PLPP3* in the corpus callosum of postmortem human brains (control, *n* = 6) by digital PCR. Data are represented as the mean ± SEM.

To examine the underpinnings for the lowered S1P levels in the corpus callosum of patients with schizophrenia, we analyzed the expression of genes involved in the metabolism of S1P (figure 2A) using the expanded sample set 2 (supplementary table S5). The outline of gene expression analysis is shown in supplementary figure S2. The expression levels of Sphingosine-1-Phosphate Lyase 1 (*SGPL1*) and Phospholipid Phosphatase 3 (*PLPP3*) showed a significantly higher (*P* = .006) and an elevated trend (*P* = .09), respectively, in the corpus callosum of patients with schizophrenia compared with controls (figure 2B). We further examined the correlation of *SGPL1* or *PLPP3* expression level with RNA integrity number (RIN), as the RIN in the corpus callosum of patients with schizophrenia differed significantly from that of the controls. There was no significant correlation between RIN values and the expression of *SGPL1* or *PLPP3* (supplementary figure S5).

The S1P content and *PLPP3* expression level displayed a trend toward negative correlation in the corpus callosum (*P* = .085), while S1P content and *SGPL1* expression were not correlated (figure 2C). Interestingly, the absolute transcript expression level of *PLPP3* was approximately 5 times higher than that of *SGPL1* in the corpus callosum (control samples) (figure 2D). This suggests that, rather than by increased *SGPL1* expression, an S1P-degrading process augmented by elevated *PLPP3* expression may, at least in part, be responsible for the lowered S1P content in the corpus callosum of patients with schizophrenia.

In the BA8, the transcript expression level of *SGPL1* or *PLPP3* was also significantly higher in the schizophrenia group than in the controls (supplementary figure S6A). The expression level of *SGPL1* or *PLPP3* exhibited no correlation with S1P content but showed a negative correlation with the RIN (supplementary figures S6B and S6C); RIN values were lower in patients with schizophrenia than in controls (supplementary table S5). Therefore, a higher expression of *SGPL1* and *PLPP3* in the BA8 of patients with schizophrenia may have stemmed from a lower RIN or associated phenomena.

### Expression of Genes for Sphingosine-1-Phosphate Receptors Is Elevated in the Corpus Callosum of Patients With Schizophrenia

**Fig. 3.**
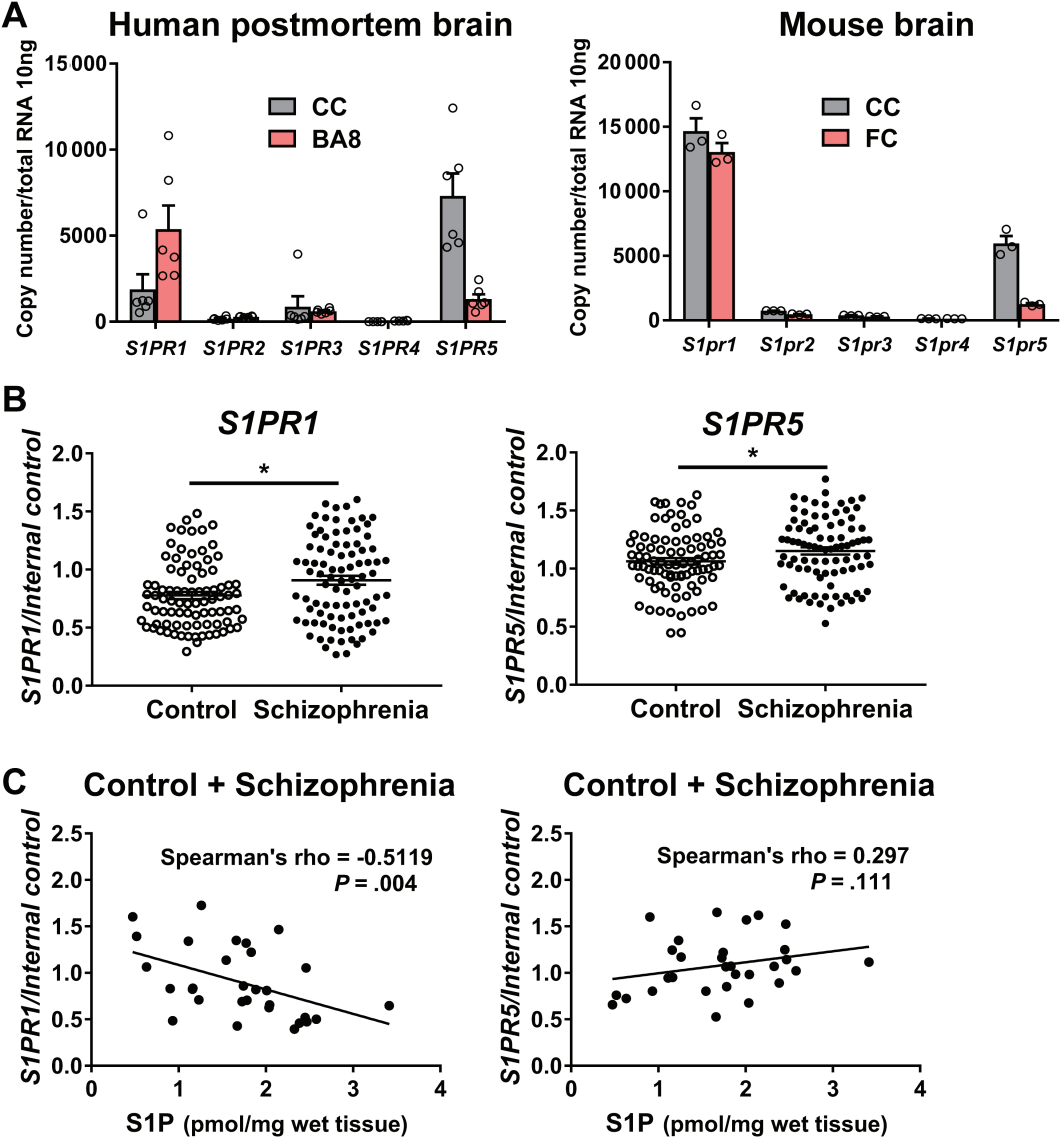
Transcript expression levels of the genes coding for S1P receptors. (A) Absolute quantification of the expression levels of genes for S1P receptors (*S1PR/S1pr1-5*) in postmortem human (control, *n* = 6) and mouse brains (C57/BL6J, *n* = 3) by digital PCR. (B) Transcript expression levels for S1P receptors in the corpus callosum of patients with schizophrenia (*n* = 91) and controls (*n* = 90). The data were normalized with the geometric mean of the 2 internal control genes (*GAPDH* and *B2M*) and are represented as the mean ± SEM. *P < .05. Difference between 2 groups was analyzed by Mann-Whitney *U* test. (C) Correlation between S1P content and transcript expression levels for *S1PR1* and *S1PR5* in the corpus callosum of patients with schizophrenia and controls (Spearman’s rank correlation coefficient). CC, corpus callosum; BA8, Brodmann area 8; FC, frontal cortex.

We then explored whether a compensatory mechanism is evoked following the lowered S1P levels for the upregulation of genes for S1P receptors. There are 5 S1P receptor genes (*S1PR1-5*), and 4 among them (*S1PR1*, *S1PR2*, *S1PR3*, and *S1PR5*) are expressed in the central nervous system (CNS).^24^Digital PCR analysis showed that absolute transcript expression of *S1PR1*/*S1pr1* and *S1PR5*/*S1pr5* was abundant in the corpus callosum and frontal cortex of both humans and mice compared with that of the other receptor subtype genes (figure 3A). Consistent with a previous literature,^24^ the expression of *S1PR4*/*S1pr4* was not detectable in our analysis. In humans, *S1PR5* expression was highest in the corpus callosum, while *S1PR1* expression was highest in the BA8 (figure 3A). In mice, *S1pr1* showed the highest expression in both the corpus callosum and the frontal cortex (figure 3A).

Next, we examined the gene expression levels of 4 S1P receptors in the human corpus callosum samples (supplementary figure S2). Expression of both *S1PR1* and *S1PR5* was also significantly higher in patients with schizophrenia than in controls (*P* = .02 and *P* = .03, respectively, figure 3B). We found a negative correlation between *S1PR5* expression level and the RIN in the corpus callosum (supplementary figure S5), indicating that a lower RIN or associated phenomena may contribute to elevated *S1PR5* expression in the corpus callosum of patients with schizophrenia. The *S1PR1* expression level was positively correlated with the RIN in the corpus callosum. However, the meaning of this weak correlation is unknown, because *S1PR1* expression was higher in patients with schizophrenia with lower RIN values (supplementary figure S5). Importantly, a negative correlation was observed between *S1PR1* expression level and S1P content in the corpus callosum (figure 3C). These results suggest that the higher expression of *S1PR1* in the corpus callosum of patients with schizophrenia might have stemmed from the lowered S1P content resulting from a compensatory mechanism.

In the BA8, higher expression of *S1PR1*, but not *S1PR5*, was found in patients with schizophrenia than in controls (supplementary figure S6A). The transcript expression level of *S1PR1* showed no correlation with S1P content and displayed a negative correlation with the RIN (supplementary figures S6B and S6C). Therefore, lower RIN values or associated phenomena may be involved in higher *S1PR1* expression in the BA8 of patients with schizophrenia.

## Discussion

To our knowledge, we were the first to demonstrate that S1P levels were significantly reduced in the corpus callosum of patients with schizophrenia. We also showed that the lowered S1P level was accompanied by an increase in the expression of gene(s) for S1P-degrading enzyme(s) PLPP3 (and SGPL1). This suggests that the upregulation of the degradation process of S1P may contribute to the lowered S1P content in the corpus callosum of patients with schizophrenia. The corpus callosum contained approximately 9 times more S1P than the BA8 (figures 1B and 1C), suggesting it is susceptible to an altered S1P metabolism.

Although schizophrenia and bipolar disorder share a genetic basis, a previous study revealed lower fatty acids levels from the skin in patients with schizophrenia, but not in those with bipolar disorder.^40^Our findings support the possibility that schizophrenia may differ from major depressive disorder and bipolar disorder with regard to biomolecular events and lowered S1P content. Lowered S1P content is involved in neuroinflammation,^41^ reduction of white matter,^4^ impairment of the blood-brain barrier,^42^ and dysregulation of autophagy,^43^ which are observed in schizophrenia.

The brain contains the highest concentration of S1P in the body.^44^ Double-knockout mice of *Sphk1/Sphk2*, whose gene products are necessary for S1P synthesis in the brain, displayed a drastic reduction of S1P content, embryonic lethality, and severe neurogenesis defects.^45–47^ This indicates S1P plays a crucial role in brain development and neural function. Furthermore, the differentiation of oligodendrocytes from induced pluripotent stem cells was impaired in patients with schizophrenia.^48^ Thus, lower S1P content in the corpus callosum may contribute to white-matter abnormalities reported in patients with schizophrenia. In relation to our findings, a previous clinical study reported lower levels of plasma S1P in patients with schizophrenia without drug intake.^49^

S1P also acts as an intracellular inhibitor of histone-deacetylase (HDAC) 1 and 2.^50^ Higher expression of *HDAC1/HDAC2* (*Hdac2*) was reported in patients with schizophrenia and schizophrenia model rats.^51–54^ Furthermore, mice overexpressing *Hdac1* in the prefrontal cortex displayed working-memory impairment.^53,55^ Therefore, lowered S1P levels may contribute to schizophrenia pathophysiology through the upregulation of HDAC1 and HDAC2 activities.

We found elevated levels of Cer and doxmeCer in the BA8. A previous study reported a higher Cer level in the prefrontal cortex of patients with schizophrenia,^56^ which is consistent with our results. Ceramide is a fatty-acid-acylated form of sphingolipid, while S1P is a base form. A differential metabolism for base forms and fatty-acid-acylated forms of sphingolipids, depending on brain region, has been reported.^57^ Therefore, a higher level of Cer in the BA8 may also contribute to schizophrenia pathophysiology, eg, through apoptotic dysregulation.^58,59^

There were several limitations to our study: (1) it remains elusive whether reduced S1P content is the primary cause and the upregulation of S1P receptor(s) is its consequence or vice versa; (2) if reduced S1P content is the primary event, the upstream mechanism remains to be delineated from genetic and epigenetic changes of genes for S1P metabolism; and (3) our gene-expression data were not corrected for multiple comparisons. Further studies using larger samples are required to validate our findings.

We have provided evidence suggesting an altered S1P-mediated signaling pathway elicited by lower S1P content may underlie the “myelin pathology” of a schizophrenia subset. Given that multiple agents have been developed against S1P receptors,^60^ our findings support a rationale that S1P receptors should be considered as a novel therapeutic target for schizophrenia. Further studies are warranted to elucidate the detailed mechanistic role of S1P and the cause of quantitative S1P changes in patients with schizophrenia.

## Funding

This work was supported by the Japan Society for the Promotion of Science (JSPS) KAKENHI (grant numbers 15K19754 and 18K15501 to K.E. and grant number 20K20388 to T.Y.), by the Grant-in-Aid for Scientific Research on Innovative Areas from the Ministry of Education, Culture, Sports, Science and Technology (MEXT), Japan (grant number JP19H05435 to T.Y.), by the AMED-CREST from the Japan Agency for Medical Research and Development (AMED) (grant number JP19gm0910004 to T.Y.).

## Supplementary Material

sbaa052_suppl_Supplementary_Material
