# Supplementary material for: Evidence for Altered Metabolism of Sphingosine-1-Phosphate in the Corpus Callosum of Patients with Schizophrenia
Source: Schizophr Bull. 2020 Apr 29;46(5):1172–81. doi: 10.1093/schbul/sbaa052 (PMC7505171; doi:10.1093/schbul/sbaa052)
Supplement: sbaa052_suppl_Supplementary_Material [file sbaa052_suppl_supplementary_material.docx]

# Supplementary Information:

**Evidence for Altered Metabolism of Sphingosine-1-phosphate in the Corpus Callosum of Patients with Schizophrenia**

**Kayoko Esaki^1^, Shabeesh Balan^1^, Yoshimi Iwayama^1,2^, Chie Shimamoto-Mitsuyama^1^, Yoshio Hirabayashi^3^, Brian Dean^4,5^, and Takeo Yoshikawa^1,*^**

^1^ Laboratory of Molecular Psychiatry, RIKEN Center for Brain Science, Saitama, Japan

^2^ Support Unit for Bio-Material Analysis, Research Division, RIKEN Center for Brain Science, Saitama, Japan

^3^ Cellular Informatics Laboratory, RIKEN Cluster for Pioneering Research, Saitama, Japan

^4^ The Florey Institute of Neuroscience and Mental Health, Howard Florey Laboratories, The University of Melbourne, Victoria, Australia

^5^ The Centre for Mental Health, Swinburne University, Victoria, Australia

Running title: Sphingosine-1-phosphate in Schizophrenia

*Corresponding author:

Takeo Yoshikawa, MD, PhD

Laboratory for Molecular Psychiatry

RIKEN Center for Brain Science

2-1 Hirosawa, Wako-city, Saitama 351-0198, Japan

Tel: +81(0) 48 467 5968

Fax: +81(0) 48 467 7462

E-mail: takeo.yoshikawa@riken.jp

**Supplementary Materials and Methods**

***Internal standard materials for* *sphingolipid analysis***

The internal standard mixture (ceramide/sphingoid internal standard mixture I), d-erythro-sphinganine-d7, d-erythro-sphingosine-d7, d-erythro-sphingosine-d7-1-phosphate, 1-deoxy-sphinganine (doxSA, m18:0), 1-deoxy-sphinganine-d3 (m18:0), 1-deoxy-sphingosine (doxSO, m18:1), 1-deoxymethyl-sphinganine (doxmeSA, m17:0), 1-deoxymethyl-sphinganine-d5 (m17:0), 1-deoxymethyl-sphingosine (doxmeSO, m17:1), N-lauroyl-dihydroceramide (C12DHCer), N-C12–1-deoxy-dihydroceramide (C12doxDHCer), N-C12–1-deoxy-ceramide (C12doxCer), N-C12-1-deoxymethyl-dihydroceramide (C12doxmeDHCer), and N-C12-1-deoxymethyl-ceramide (C12doxmeCer) were purchased from Avanti Polar Lipids (Alabaster, AL, USA).

***Lipid extraction and analysis***

In total, 400 μl of cold phosphate-buffered saline (PBS) and 10-20 mg of tissue samples were homogenized by sonication. After the addition of 1.5 ml of chloroform/methanol (1:2, v/v), the homogenate was added to 25 pmol of the internal standard mixture and incubated at 48 °C overnight. After the addition of 150 μl of 1 M potassium hydroxide in methanol, the mixture was incubated for 2 h at 37 °C and neutralized by adding glacial acetic acid. For the analysis of base-form sphingolipids (sphingoid base: sphinganine [SA], sphingosine [SO], sphingosine-1-phosphate [S1P], doxSA, doxSO, doxmeSA, and doxmeSO in figure 1A), the samples were centrifuged at 1,500 *g* for 10 min to obtain the supernatant. The residue was re-extracted with 1 ml of chloroform/methanol (2:1, v/v) and centrifuged at 1,500 *g* for 10 min. The two supernatants were combined for analysis. For the analysis of fatty-acid-acylated-form sphingolipids (dihydroceramide [DHCer], ceramide [Cer], 1-deoxy-dihydroceramide [doxDHCer], 1-deoxy-ceramide [doxCer], 1-deoxymethyl-dihydroceramide [doxmeDHCer], 1-deoxymethyl-ceramide [doxmeCer], lactosylceramide [LacCer], hexosylceramide [HexCer] and sphingomyelin [SM] in figure 1A), 1 ml of chloroform and 2 ml of water were added to the samples; the samples were vortexed well and centrifuged at 1,500 *g* rpm for 10 min to obtain a two-phase system, the aqueous top phase and the organic bottom phase, from which lipids were extracted. The top phase was re-extracted with an additional 1 ml of chloroform and centrifugation at 1,500 *g* for 10 min, then the organic phases were combined. These lipid extracts were dried under a N_2_ stream and stored at -20 °C until use. The dried residue was dissolved in the mobile phase solvent immediately before liquid chromatography electrospray ionization tandem mass spectrometry (LC-ESI-MS/MS) analysis using the Nexera X2 HPLC system (Shimadzu, Kyoto, Japan) and QTRAP 4500 (AB SCIEX, Framingham, MA).^1,2^

***Animals***

Male C57BL/6J mice (6 weeks old) were purchased from Charles River Laboratories (Yokohama, Japan) and maintained in a 12-h light/dark cycle with unlimited access to food and water. Vehicle PBS (10 ml/day/kg body weight), haloperidol (0.1 mg/kg body weight; Dainippon Sumitomo Pharma Co., Ltd., Osaka, Japan) or risperidone (0.2 mg/kg body weight; Kobayashikako Co., Ltd., Fukui, Japan) were intraperitoneally (i.p.) administered for 4 weeks. Twenty-four hours after the last injection, the mice (vehicle, *n* = 10; haloperidol, *n* = 10; risperidone, *n* = 10) were euthanized by cervical dislocation and the brains excised as quickly as possible. The frontal cortex and corpus callosum were isolated on an ice-cooled glass plate, flash-frozen in liquid N_2_, and stored at -80 °C until gene expression or sphingolipid analyses were performed. The experimental procedure was approved by the Animal Ethics Committee at RIKEN.

***Gene expression analysis***

Total RNA was extracted from the mice and human brain samples using the QIAGEN miRNeasy Mini kit (QIAGEN, Hilden, Germany) and reverse transcribed using the SuperScript VILO Master Mix (Invitrogen, Grand Island, NY, USA). Real-time reverse transcription polymerase chain reaction (RT-PCR) analysis was performed with TaqMan^®^ Gene Expression Assays (supplementary table S8) using TaqMan^®^ Gene Expression Master Mix (Cat# 4370074, Applied Biosystems, Foster City, CA, USA) according to the manufacturer’s recommendations in the QuantStudio™ 12K Flex Real-Time PCR System‎ (ThermoFisher, Waltham, MA, USA). All real-time RT-PCR reactions were performed in triplicate, based on the standard curve method, and captured using the QuantStudio™ 12K Flex Software v1.2.3 (ThermoFisher). Expression of the selected target genes were tested by normalizing with *GAPDH* as reference gene. Only those genes which showed significant (or a trend for) differences were further proceeded with quantification by normalizing to the geometric mean of *GAPDH* and *B2M*. The overall flow of gene expression analysis is shown in supplementary figure S2.

Absolute quantitative analysis of mRNAs for the genes coding for S1P-degrading enzymes or S1P receptor subtypes was conducted using TaqMan^®^ Gene Expression Assays (Applied Biosystems, Foster City, CA, USA) in the QuantStudio™ 3D digital PCR system (ThermoFisher) with samples of the corpus callosum and BA8/frontal cortex from human controls (*n* = 6) and C57BL/6J mice (*n* = 3). All absolute quantification data were captured using QuantStudio™ 3D AnalysisSuite™ software (ThermoFisher).

**References**

**1.** Esaki K, Sayano T, Sonoda C, et al. L-Serine Deficiency Elicits Intracellular Accumulation of Cytotoxic Deoxysphingolipids and Lipid Body Formation. *J Biol Chem* 2015;290:14595-14609.

**2.** Shaner RL, Allegood JC, Park H, et al. Quantitative analysis of sphingolipids for lipidomics using triple quadrupole and quadrupole linear ion trap mass spectrometers. *J Lipid Res* 2009;50:1692-1707.

**Supplementary Table S1.** Clinical data of patients with schizophrenia

| ID | Suicide | Cause of death | Antipsychotic drug | CPz eq. |
| --- | --- | --- | --- | --- |
| 1 | No | Ruptured aneurysm (abdomen) | Fluphenazine decanoate Haloperidol | 550 |
| 2 | No | Respiratory failure | Clozapine | 631.75 |
| 3 | Yes | Hypovolemic shock | Risperdone | 285 |
| 4 | No | Intestinal ischemia | Fluphenazine decanoate Chlorpromazine | 1700 |
| 5 | No | Chronic cardiac failure | Fluphenazine decanoate Chlorpromazine | 800 |
| 6 | Yes | CO poisoning | Drug free | N/A |
| 7 | No | Mediastinitis | Haloperidol | 160 |
| 8 | No | Cerebral amyloid angiopathy | Thioridazine | 400 |
| 9 | No | Pulmonary thromboembolism | Fluphenazine decanoate Chlopromazine | 700 |
| 10 | No | Hypothermia | Drug Free | N/A |
| 11 | Yes | Hanging | Zuclopenthixol Amisulpride | 1575 |
| 12 | No | Pneumonia | Risperidone | 600 |
| 13 | No | Pneumonia | Risperidone | 800 |
| 14 | No | Pleural metastasis | Drug Free | N/A |
| 15 | No | Ischemic heart disease | Drug Free | N/A |

CPz eq., chlorpromazine equivalents.

**Supplementary Table S2.** Clinical data of patients with major depressive disorder

| ID | Suicide | Cause of death | Antidepressant drug | CPz eq. | Fluox eq. |
| --- | --- | --- | --- | --- | --- |
| 1 | Yes | Overdose of Melleril |  |  |  |
| 2 | Yes | Mixed drug toxicity |  | 268 |  |
| 3 |  | Acute alcohol toxicity | Prothiaden | 50 | 41.2 |
| 4 | Yes | Hanging | Venlafaxine |  | 40.2 |
| 5 | Yes | Hanging |  |  |  |
| 6 | Yes | Drowning |  |  |  |
| 7 | Yes | CO poisoning |  |  |  |
| 8 | Yes | Hanging | Clomipramine Venlafaxine |  | 51.7 |
| 9 | Yes | Hanging |  |  |  |
| 10 | No | Pulmonic embolism  Left calf deep venous thrombosis | Amitriptyline |  | 16.3 |
| 11 | Yes | Hanging |  |  |  |
| 12 | ?Yes | Mixed drug toxicity |  |  |  |
| 13 | Yes | Asphyxia/CO poisoning |  |  |  |
| 14 | Yes | Toxicity to Quetiapine | Mirtazapine | 610 | 23.6 |
| 15 | Yes | Hanging |  |  |  |

CPz eq., chlorpromazine equivalents; Fluox eq., fluoxetine equivalents.

**Supplementary Table S3.** Clinical data of patients with bipolar disorder

| ID | Suicide | Cause of death | Antipsychotic drug | CPz eq. | Antidepressant Drug | Fluox eq. | Mood Stabilsers | Lithium eq. |
| --- | --- | --- | --- | --- | --- | --- | --- | --- |
| 1 | No | Ischemic heart disease  Coronary artery disease |  |  | Venlafaxine | 80.3 | Sodium valproate | 650 |
| 2 | No | Cardiomegaly |  |  |  |  |  |  |
| 3 | No | Acute myocardial infarction |  |  |  |  | Sodium valproate | 3437 |
| 4 | No | Ischemic heart disease  Coronary artery disease |  |  |  |  | Lithium | 500 |
| 5 | No | Ischemic heart disease |  |  |  |  |  |  |
| 6 | No | Aspiration of food | Fluphenazine decanoate | 166 |  |  |  |  |
| 7 | Yes | CO poisoning |  |  | Paroxetine | 23.5 | Lithium | 1500 |
| 8 | No | Aortal rupture (Natural) |  |  |  |  | Lithium | 1000 |
| 9 | No | Unascertained | Chlorpromazine | 300 |  |  | Lithium  Carbamazepine | 1600 |
| 10 | No | Combined drug toxicity | Fluphenazine decanoate | 100 | Dothiepin HCl | 38.7 |  |  |
| 11 | Yes | CO poisoning | Zuclopenthixol | 300 |  |  |  |  |
| 12 | Yes | Hanging |  |  |  |  |  |  |
| 13 | No | Chronic cholecystitis | Zyprexia | 675 |  |  | Sodium valproate | 275 |
| 14 | Yes | CO poisoning |  |  |  |  | Carbamazepine | 400 |
| 15 | No | Aspiration of food | Risperidone | 254 |  |  | Sodium valproate | 1375 |

CPz eq., chlorpromazine equivalents; Fluox eq., fluoxetine equivalents; Lithium eq., lithium equivalents.

**Supplementary Table S4.** Clinical data of controls

| ID | Suicide | Cause of death |
| --- | --- | --- |
| 1 | No | Aortic aneurysm |
| 2 | No | Ischemic heart disease |
| 3 | No | Thoracic aorta disease |
| 4 | No | Coronary artery atherosclerosis |
| 5 | No | Coronary artery atherosclerosis |
| 6 | No | Coronary artery atheroma |
| 7 | No | Coronary artery atheroma |
| 8 | No | Cardiomegaly, ischemic heart disease and coronary artery disease |
| 9 | No | Mitral valve prolapse |
| 10 | No | Hypertensive heart disease |
| 11 | No | Acute myocardial infarct |
| 12 | No | Pulmonary embolus |
| 13 | No | Cardiogenic shock |
| 14 | No | Pericardial tamponade |
| 15 | No | Cardiomegaly and ischemic heart disease |

**Supplementary Table S5.** Characteristics of postmortem brain sample set 2 for gene expression analysis

|  | Control *n* = 90 | Schizophrenia *n* =91 | *P* value |
| --- | --- | --- | --- |
| Age at death (years) | 47.6 ± 16.5 | 45.7 ± 17.5 | 0.39 |
| Sex (male/female) | 70/20 | 68/23 | 0.73 |
| Duration of illness (years) |  | 18.1 ± 14.4 |  |
| Postmortem interval (hours) | 42.0 ± 14.0 | 41.5 ± 14.0 | 0.87 |
| Brain tissue pH | 6.34 ± 0.20 | 6.29 ± 0.21 | 0.10 |
| RIN (corpus callosum) | 6.3 ± 0.8 | 5.7 ± 1.3 | < 0.001 |
| RIN (Brodmann area 8) | 7.4 ± 0.7 | 6.6 ± 1.4 | < 0.001 |

The values represent the mean ± SD. Differences between two groups were analyzed by Mann-Whitney U test. RIN, RNA integrity number.

**Supplementary Table S6.** Levels of sphingolipids in the corpus callosum from the subjects with schizophrenia and controls

| N-acyl chain | | C14 | C16 | C18 | C18:1 | C20 | C20:1 | C22 | C22:1 | C24 | C24:1 | C26 | C26:1 | SUM |
| --- | --- | --- | --- | --- | --- | --- | --- | --- | --- | --- | --- | --- | --- | --- |
| DH Cer | CON | 0.02 ± 0.00 | 0.05 ± 0.01 | 1.89 ± 0.20 | 0.01 ± 0.00 | 0.01 ± 0.00 | 0.00 ± 0.00 | 0.05 ± 0.01 | 0.02 ± 0.00 | 0.60 ± 0.08 | 0.69 ± 0.07 | 0.07 ± 0.01 | 0.21 ± 0.02 | 3.63 ± 0.35 |
|  | SCZ | 0.02 ± 0.00 | 0.05 ± 0.01 | 2.18 ± 0.49 | 0.02 ± 0.00 | 0.01 ± 0.00 | 0.01 ± 0.00 | 0.08 ± 0.02 | 0.02 ± 0.01 | 0.68 ± 0.17 | 0.98 ± 0.27 | 0.06 ± 0.01 | 0.21 ± 0.03 | 4.32 ± 0.98 |
|  | *P* | > 0.99 | > 0.99 | 0.74 | 0.98 | 0.67 | 0.90 | 0.65 | 0.49 | 0.77 | 0.90 | 0.41 | 0.56 | 0.68 |
| LacCer | CON | 0.43 ± 0.05 | 4.32 ± 0.73 | 27.80 ± 1.92 | 0.20 ± 0.05 | 0.93 ± 0.17 | 0.18 ± 0.03 | 2.71 ± 0.32 | 0.56 ± 0.08 | 6.39 ± 0.58 | 32.52 ± 2.65 | 1.27 ± 0.08 | 6.48 ± 0.48 | 83.80 ± 5.48 |
|  | SCZ | 0.33 ± 0.04 | 3.18 ± 0.39 | 25.44 ± 2.56 | 0.15 ± 0.02 | 0.90 ± 0.11 | 0.27 ± 0.07 | 2.63 ± 0.31 | 0.52 ± 0.06 | 6.97 ± 0.63 | 34.71 ± 3.25 | 1.31 ± 0.10 | 6.81 ± 0.62 | 83.24 ± 7.16 |
|  | *P* | 0.19 | 0.22 | 0.48 | 0.36 | 0.62 | 0.65 | 0.87 | > 0.99 | 0.59 | 0.71 | 0.51 | 0.84 | > 0.99 |
| HexCer | CON | 0.00 ± 0.00 | 0.02 ± 0.00 | 1.01 ± 0.12 | 0.01 ± 0.00 | 0.11 ± 0.02 | 0.04 ± 0.01 | 0.37 ± 0.06 | 0.07 ± 0.01 | 2.29 ± 0.37 | 7.84 ± 1.14 | 0.29 ± 0.06 | 1.86 ± 0.34 | 13.93 ± 2.08 |
|  | SCZ | 0.00 ± 0.00 | 0.02 ± 0.00 | 0.97 ± 0.13 | 0.01 ± 0.00 | 0.10 ± 0.01 | 0.05 ± 0.02 | 0.36 ± 0.05 | 0.07 ± 0.01 | 2.07 ± 0.31 | 7.14 ± 1.05 | 0.23 ± 0.04 | 1.61 ± 0.25 | 12.63 ± 1.80 |
|  | *P* | 0.71 | 0.87 | 0.74 | 0.40 | 0.97 | 0.71 | > 0.99 | 0.90 | 0.68 | 0.71 | 0.62 | 0.84 | 0.74 |
| DoxCer | CON | 0.22 ± 0.11 | 0.26 ± 0.04 | 12.63 ± 1.20 | 0.12 ± 0.01 | 0.15 ± 0.04 | N.D. | 0.50 ± 0.07 | N.D. | 6.40 ± 0.71 | 1.87 ± 0.32 | 0.57 ± 0.15 | 4.06 ± 0.63 | 25.25 ± 2.44 |
|  | SCZ | 0.11 ± 0.03 | 0.25 ± 0.05 | 11.06 ± 1.61 | 0.16 ± 0.02 | 0.13 ± 0.02 | N.D. | 0.46 ± 0.07 | N.D. | 5.23 ± 0.89 | 1.76 ± 0.38 | 0.47 ± 0.17 | 3.55 ± 0.94 | 22.32 ± 3.67 |
|  | *P* | 0.40 | 0.53 | 0.19 | 0.24 | > 0.99 |  | 0.71 |  | 0.11 | 0.50 | 0.31 | 0.28 | 0.20 |
| Dox DHCer | CON | N.D. | 0.99 ± 0.27 | 4.33 ± 0.35 | 0.43 ± 0.10 | 0.64 ± 0.08 | N.D. | 8.46 ± 1.04 | 0.13 ± 0.01 | 99.44 ± 12.70 | 37.69 ± 6.57 | 13.13 ± 2.03 | 55.77 ± 7.43 | 220.92 ± 26.04 |
|  | SCZ | N.D. | 0.31 ± 0.05 | 4.40 ± 0.38 | 0.45 ± 0.10 | 0.83 ± 0.20 | N.D. | 7.68 ± 0.81 | 0.18 ± 0.04 | 92.53 ± 9.92 | 30.58 ± 2.98 | 9.53 ± 1.05 | 45.16 ± 4.60 | 191.50 ± 16.65 |
|  | *P* |  | 0.03 | 0.86 | 0.94 | 0.83 |  | 0.72 | 0.80 | 0.93 | 0.77 | 0.21 | 0.39 | 0.57 |
| Doxme  DHCer | CON | N.D. | 0.07 ± 0.01 | 0.40 ± 0.03 | 0.17 ± 0.01 | N.D. | N.D. | 0.11 ± 0.02 | N.D. | 0.31 ± 0.05 | 0.61 ± 0.11 | 0.25 ± 0.03 | 0.58 ± 0.12 | 1.96 ± 0.26 |
|  | SCZ | N.D. | 0.05 ± 0.00 | 0.40 ± 0.05 | 0.12 ± 0.04 | N.D. | N.D. | 0.07 ± 0.01 | N.D. | 0.39 ± 0.06 | 0.64 ± 0.11 | 0.26 ± 0.04 | 0.39 ± 0.04 | 1.79 ± 0.15 |
|  | *P* |  | 0.59 | 0.78 | 0.62 |  |  | 0.003 |  | 0.10 | 0.64 | 0.69 | 0.75 | > 0.99 |

The values represent the mean ± SEM (*n* = 15 each). The concentrations are shown as pmol/mg wet tissue [dihydroceramide (DHCer) and lactosylceramide (LacCer)], nmol/mg wet tissue [hexosylceramide (HexCer)], or fmol/mg wet tissue [1-deoxy-ceramide (doxCer), 1-deoxy-dihydroceramide (doxDHCer), and 1-deoxymethyl-dihydroceramide (doxmeDHCer)]. Differences between two groups were analyzed by Mann-Whitney U test. CON, control; SCZ, schizophrenia; N.D., not detected.

**Supplementary Table S7.** Levels of sphingolipids in the Brodmann area 8 from the subjects with schizophrenia and controls

| N-acyl chain | | C14 | C16 | C18 | C18:1 | C20 | C20:1 | C22 | C22:1 | C24 | C24:1 | C26 | C26:1 | SUM |
| --- | --- | --- | --- | --- | --- | --- | --- | --- | --- | --- | --- | --- | --- | --- |
| DH Cer | CON | 0.01 ± 0.00 | 0.02 ± 0.00 | 0.23 ± 0.05 | 0.01 ± 0.00 | 0.00 ± 0.00 | 0.00 ± 0.00 | 0.01 ± 0.00 | 0.00 ± 0.00 | 0.01 ± 0.01 | 0.04 ± 0.01 | 0.00 ± 0.00 | 0.01 ± 0.00 | 0.33 ± 0.05 |
|  | SCZ | 0.01 ± 0.00 | 0.02 ± 0.00 | 0.23 ± 0.03 | 0.01 ± 0.00 | 0.00 ± 0.00 | 0.00 ± 0.00 | 0.01 ± 0.00 | 0.00 ± 0.00 | 0.02 ± 0.01 | 0.05 ± 0.01 | 0.00 ± 0.00 | 0.01 ± 0.00 | 0.36 ± 0.04 |
|  | *p* | 0.77 | 0.17 | 0.41 | 0.19 | 0.49 | 0.64 | 0.57 | 0.75 | 0.28 | 0.68 | 0.15 | 0.90 | 0.23 |
| LacCer | CON | 0.19 ± 0.03 | 1.24 ± 0.18 | 7.57 ± 1.19 | 0.12 ± 0.03 | 0.47 ± 0.07 | 0.16 ± 0.04 | 0.47 ± 0.08 | 0.09 ± 0.02 | 0.99 ± 0.17 | 6.65 ± 1.19 | 0.22 ± 0.04 | 1.59 ± 0.24 | 19.75 ± 3.09 |
|  | SCZ | 0.27 ± 0.06 | 2.62 ± 0.85 | 13.74 ± 5.43 | 0.21 ± 0.08 | 0.62 ± 0.13 | 0.45 ± 0.23 | 0.76 ± 0.19 | 0.18 ± 0.06 | 1.05 ± 0.17 | 9.84 ± 2.54 | 0.23 ± 0.05 | 1.82 ± 0.35 | 31.79 ± 9.17 |
|  | *P* | 0.65 | 0.34 | 0.62 | 0.71 | 0.59 | 0.49 | 0.53 | 0.74 | 0.90 | 0.74 | > 0.99 | 0.97 | 0.71 |
| HexCer | CON | N.D. | 0.00 ± 0.00 | 0.38 ± 0.10 | 0.09 ± 0.03 | 0.06 ± 0.02 | 0.17 ± 0.05 | 0.22 ± 0.05 | 0.06 ± 0.02 | 0.94 ± 0.25 | 4.29 ± 1.09 | 0.07 ± 0.02 | 0.92 ± 0.22 | 7.18 ± 1.83 |
|  | SCZ | N.D. | 0.00 ± 0.00 | 0.59 ± 0.31 | 0.10 ± 0.05 | 0.09 ± 0.04 | 0.24 ± 0.13 | 0.33 ± 0.15 | 0.08 ± 0.04 | 1.31 ± 0.61 | 6.06 ± 2.66 | 0.10 ± 0.05 | 1.27 ± 0.52 | 10.19 ± 4.55 |
|  | *P* |  | 0.93 | 0.74 | 0.44 | > 0.99 | 0.68 | 0.74 | 0.71 | 0.81 | 0.77 | 0.90 | 0.97 | 0.84 |
| DoxCer | CON | 2.76 ± 1.48 | N.D. | N.D. | N.D. | N.D. | 0.23 ± 0.06 | N.D. | N.D. | N.D. | 0.32 ± 0.04 | N.D. | N.D. | 1.61 ± 0.73 |
|  | SCZ | 3.60 ± 1.18 | N.D. | N.D. | N.D. | N.D. | 0.21 ± 0.04 | N.D. | N.D. | N.D. | 1.92 ± 0.86 | N.D. | 0.71 ± 0.37 | 2.89 ± 0.78 |
|  | *P* | 0.26 |  |  |  |  | 0.63 |  |  |  | 0.20 |  |  | 0.17 |
| DoxDHCer | CON | N.D. | N.D. | 6.22 ± 0.97 | 0.45 ± 0.11 | 3.75 ± 0.66 | N.D. | 19.54 ± 2.93 | N.D. | 13.41 ± 2.58 | 14.65 ± 3.13 | 1.44 ± 0.40 | 6.72 ± 2.04 | 60.25 ± 7.52 |
|  | SCZ | N.D. | N.D. | 6.80 ± 0.72 | 0.37 ± 0.03 | 3.39 ± 0.45 | N.D. | 17.89 ± 2.97 | N.D. | 18.04 ± 4.90 | 14.20 ± 3.16 | 1.93 ± 0.79 | 8.88 ± 2.83 | 65.91 ± 11.55 |
|  | *P* |  |  | 0.37 | 0.42 | 0.95 |  | 0.58 |  | 0.63 | 0.94 | 0.99 | 0.54 | 0.78 |
| Doxme DHCer | CON | N.D. | N.D. | 0.22 ± 0.04 | N.D. | N.D. | N.D. | 0.10 ± 0.03 | N.D. | 0.14 ± 0.03 | 0.25 ± 0.10 | N.D. | N.D. | 0.38 ± 0.08 |
|  | SCZ | N.D. | N.D. | 0.23 ± 0.03 | N.D. | N.D. | N.D. | 0.11 ± 0.03 | N.D. | 0.22 ± 0.10 | 0.29 ± 0.22 | N.D. | N.D. | 0.31 ± 0.07 |
|  | *P* |  |  | 0.93 |  |  |  | > 0.99 |  | 0.66 | 0.88 |  |  | 0.56 |

The values represent the mean ± SEM (*n* = 15 each). The concentrations are shown as pmol/mg wet tissue [dihydroceramide (DHCer) and lactosylceramide (LacCer)], nmol/mg wet tissue [hexosylceramide (HexCer)], or fmol/mg wet tissue [1-deoxy-ceramide (doxCer), 1-deoxy-dihydroceramide (doxDHCer), and 1-deoxymethyl-dihydroceramide (doxmeDHCer)]. Differences between two groups were analyzed by Mann-Whitney U test. CON, control; SCZ, schizophrenia; N.D., not detected.

**Supplementary Table S8.** Details of primer/probes used for gene expression analysis

| Gene symbol | Sequence (5′−3′) |
| --- | --- |
| *GAPDH* | TaqMan Probe ID Hs02758991_g1 |
| *B2M* | TaqMan Probe ID Hs99999907_m1 |
| *SPHK1* | TaqMan Probe ID Hs00184211_m1 |
| *SPHK2* | TaqMan Probe ID Hs01016543_g1 |
| *ENPP2* | TaqMan Probe ID Hs00905117_m1 |
| *SGPP1* | TaqMan Probe ID Hs00229266_m1 |
| *SGPP2* | TaqMan Probe ID Hs00544786_m1 |
| *SGPL1* | TaqMan Probe ID Hs00900722_m1 |
| *PLPP1* | TaqMan Probe ID Hs00170356_m1 |
| *PLPP2* | TaqMan Probe ID Hs00186575_m1 |
| *PLPP3* | TaqMan Probe ID Hs00170359_m1 |
| *S1PR1* | TaqMan Probe ID Hs01922614_s1 |
| *S1PR2* | probe: CACGTGGCCATTGCC/ forward: CCTCTGTCTTCAGCCTCCTG/ reverse: GCTCTTGTCGCTGCCATACA |
| *S1PR3* | TaqMan Probe ID Hs00245464_s1 |
| *S1PR5* | TaqMan Probe ID Hs00928195_s1 |


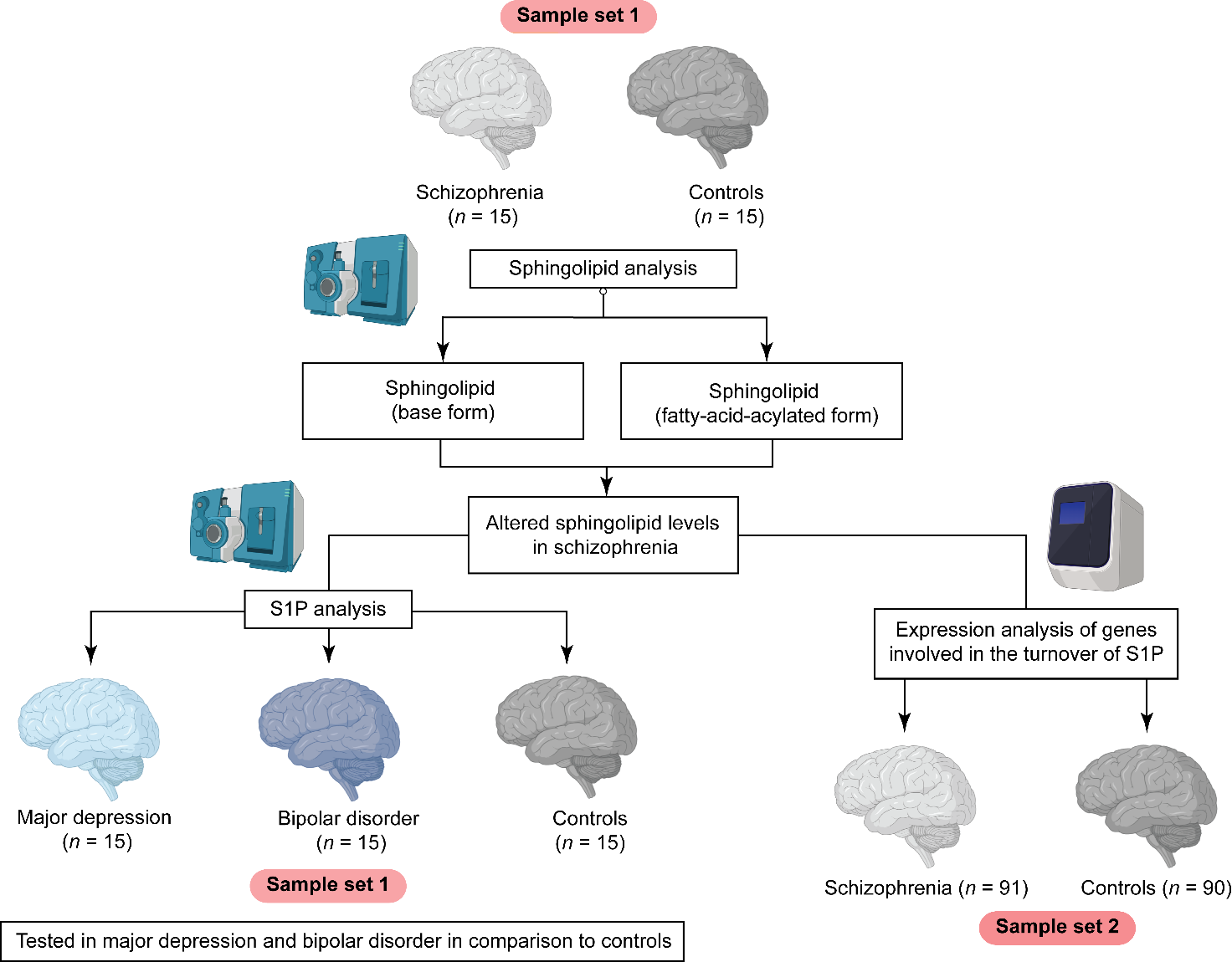


**Supplementary Figure S1. Overall flow of the experiment.**


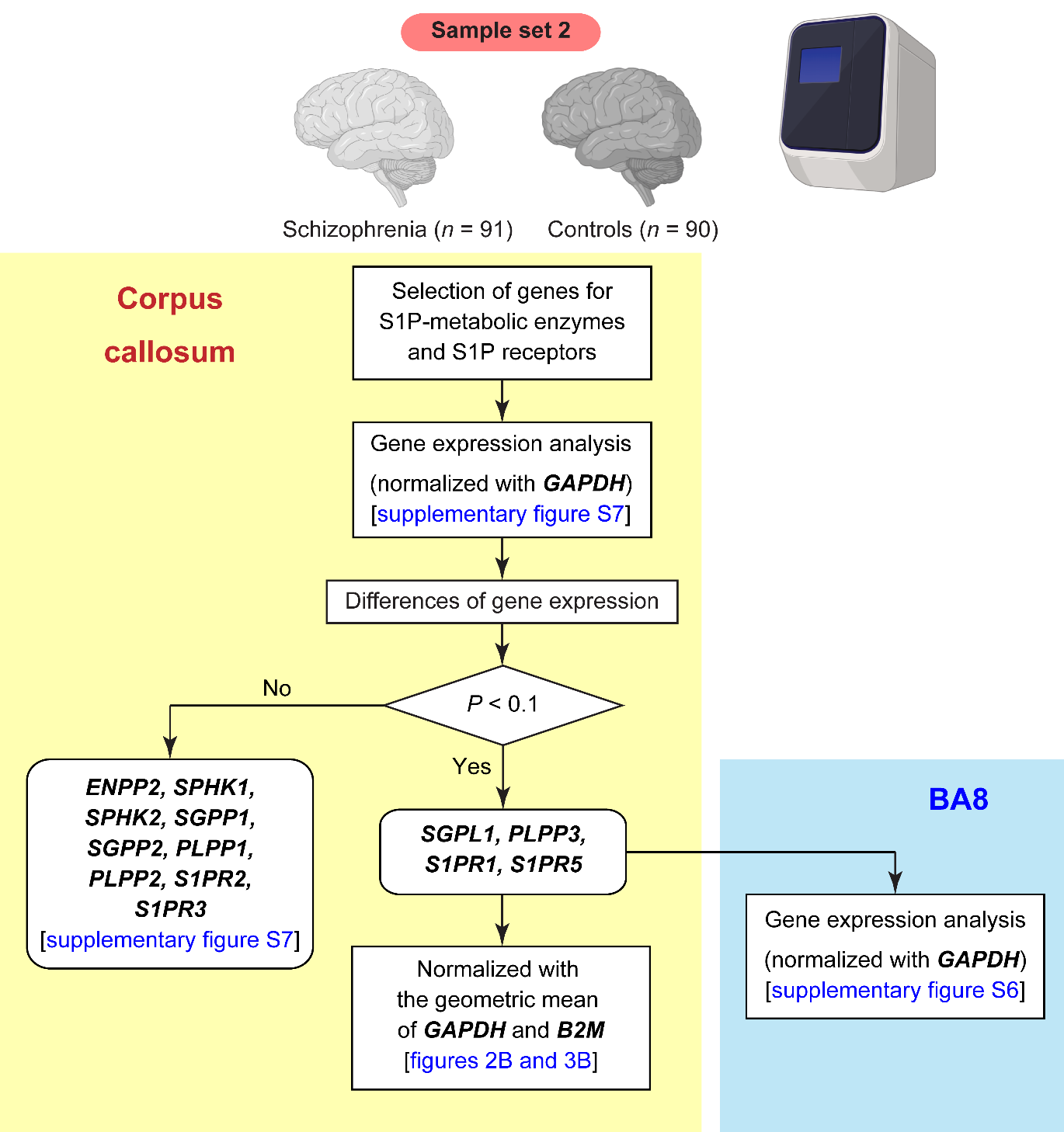


**Supplementary Figure S2. Overall flow of gene expression analysis**


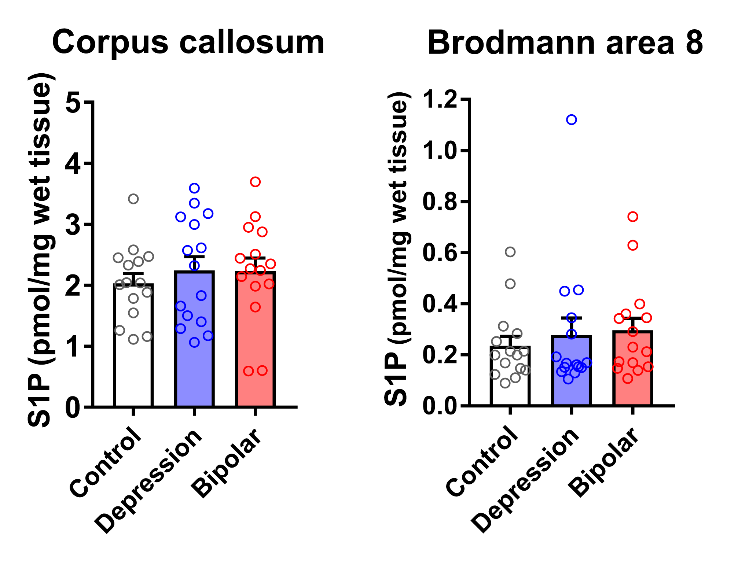


**Supplementary Figure S3. No differences in sphingosine-1-phosphate (S1P) levels were observed in the postmortem brain samples from subjects with major depressive disorder and bipolar disorder**. Lipids were extracted from the corpus callosum and Brodmann area 8 of subjects with major depressive disorder (*n* = 15), bipolar disorder (*n* = 15), and controls (*n* = 15). Then, extracted lipids were analyzed using liquid chromatography electrospray ionization tandem mass spectrometry (LC-ESI-MS/MS). Data are represented as the mean ± SEM. There were no significant differences in S1P levels of the tested groups. Differences among three groups were analyzed using the non-parametric Kruskal–Wallis one-way analysis of variance, followed by Dunnett’s test (vs. control group).


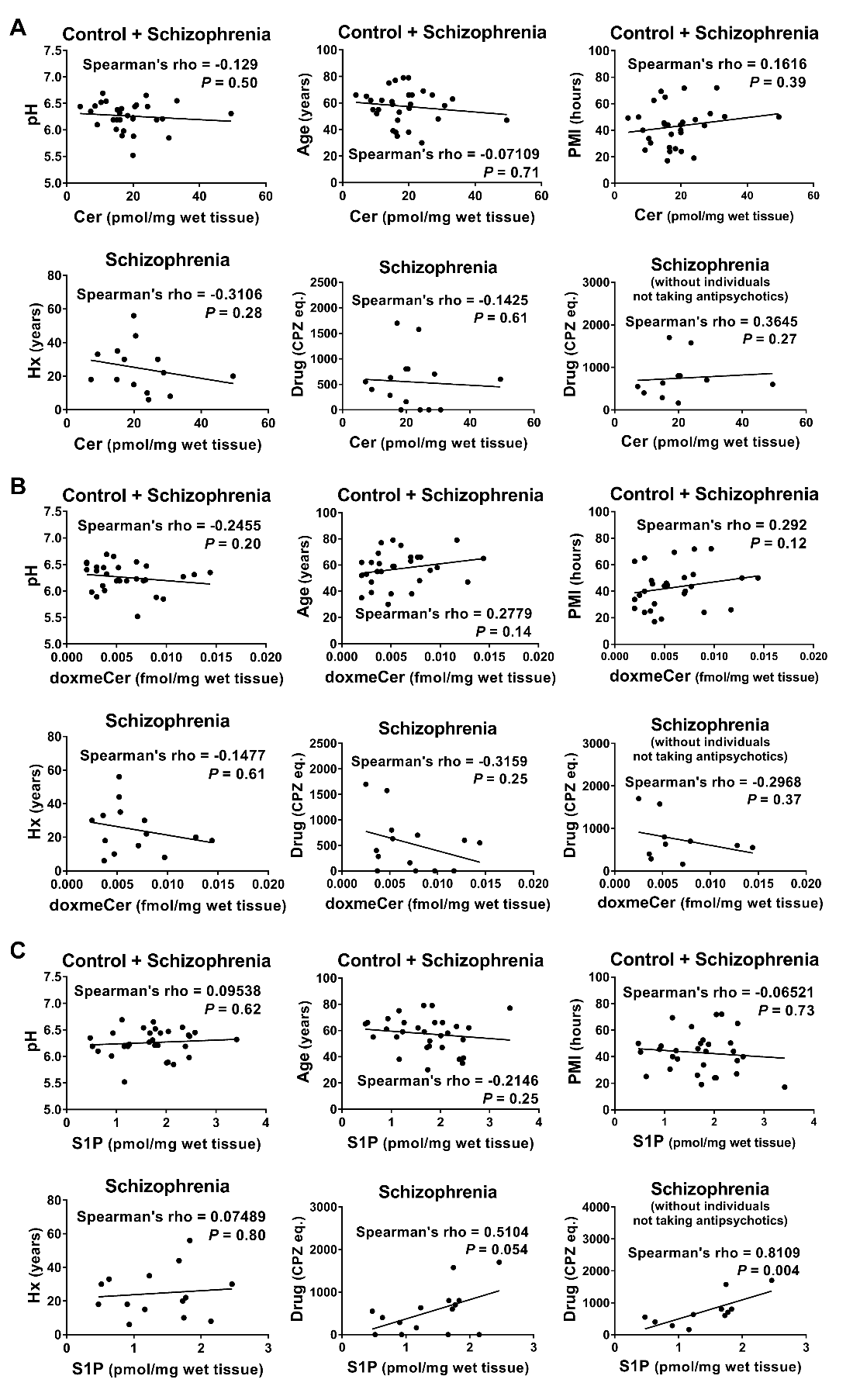


**Supplementary Figure S4. Correlation analysis of confounding factors with sphingolipid levels.** We examined the correlation of confounding factors with levels of (A) Cer and (B) doxmeCer, which included all measured fatty-acid-acylated forms, in the Brodmann area 8 or (C) S1P levels in the corpus callosum. Correlation was evaluated using Spearman’s rank correlation coefficient. Cer, ceramide; doxmeCer, 1-deoxymethyl-ceramide; PMI, postmortem interval; Hx, duration of illness; CPZ eq., chlorpromazine equivalents.


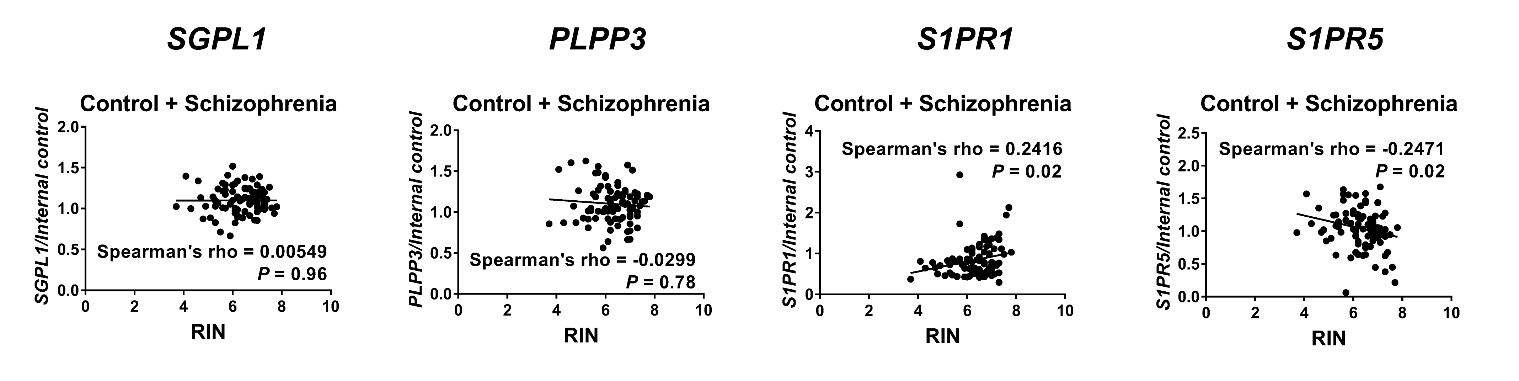


**Supplementary Figure S5. Correlations between brain tissue RNA integrity number and transcript expression level of genes coding for S1P-metabolizing enzymes and S1P receptors in the corpus callosum of subjects with schizophrenia and controls**. The expression data of genes was normalized with the geometric mean of the two internal control genes (*GAPDH* and *B2M*). Correlation was evaluated by Spearman’s rank correlation coefficient. RIN, RNA integrity number.


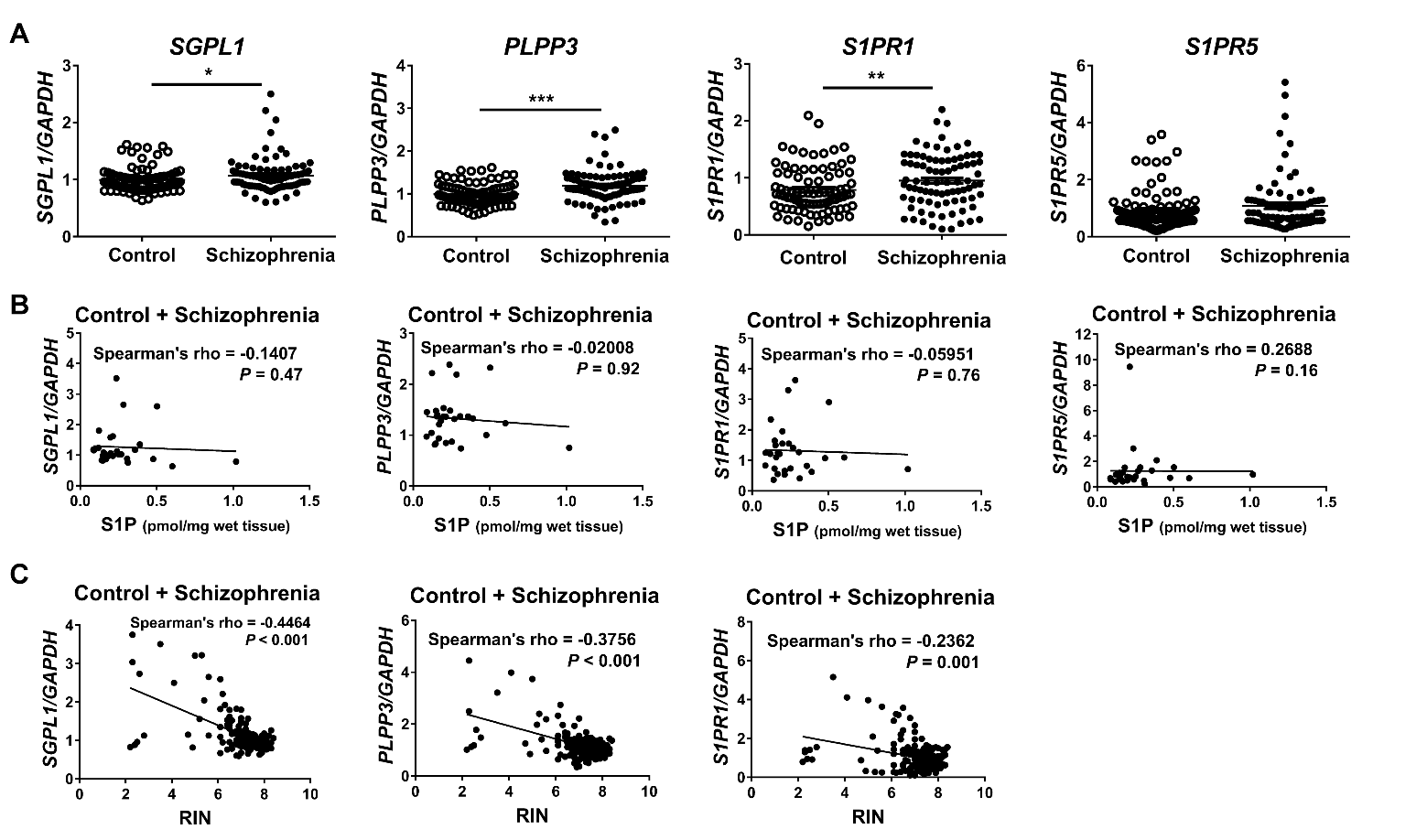


**Supplementary Figure S6. Transcript expression level of genes coding for sphingolipid metabolism-related enzymes and S1P receptors in human Brodmann area 8 tissues.** (A) Transcript expression levels of genes for S1P-metabolizing enzymes and S1P receptors in the Brodmann area 8 of subjects with schizophrenia (*n* = 91) and controls (*n* = 90), normalized with *GAPDH*. Data are presented as the mean ± SEM. **P* < 0.05, ***P* < 0.01, ****P* < 0.001. Differences between two groups were analyzed by Mann-Whitney U test. Correlations between transcript expression levels of S1P-related genes and (B) S1P levels and (C) RNA integrity number in the Brodmann area 8 of subjects with schizophrenia and controls. Correlation was evaluated by Spearman’s rank correlation coefficient. RIN, RNA integrity number.


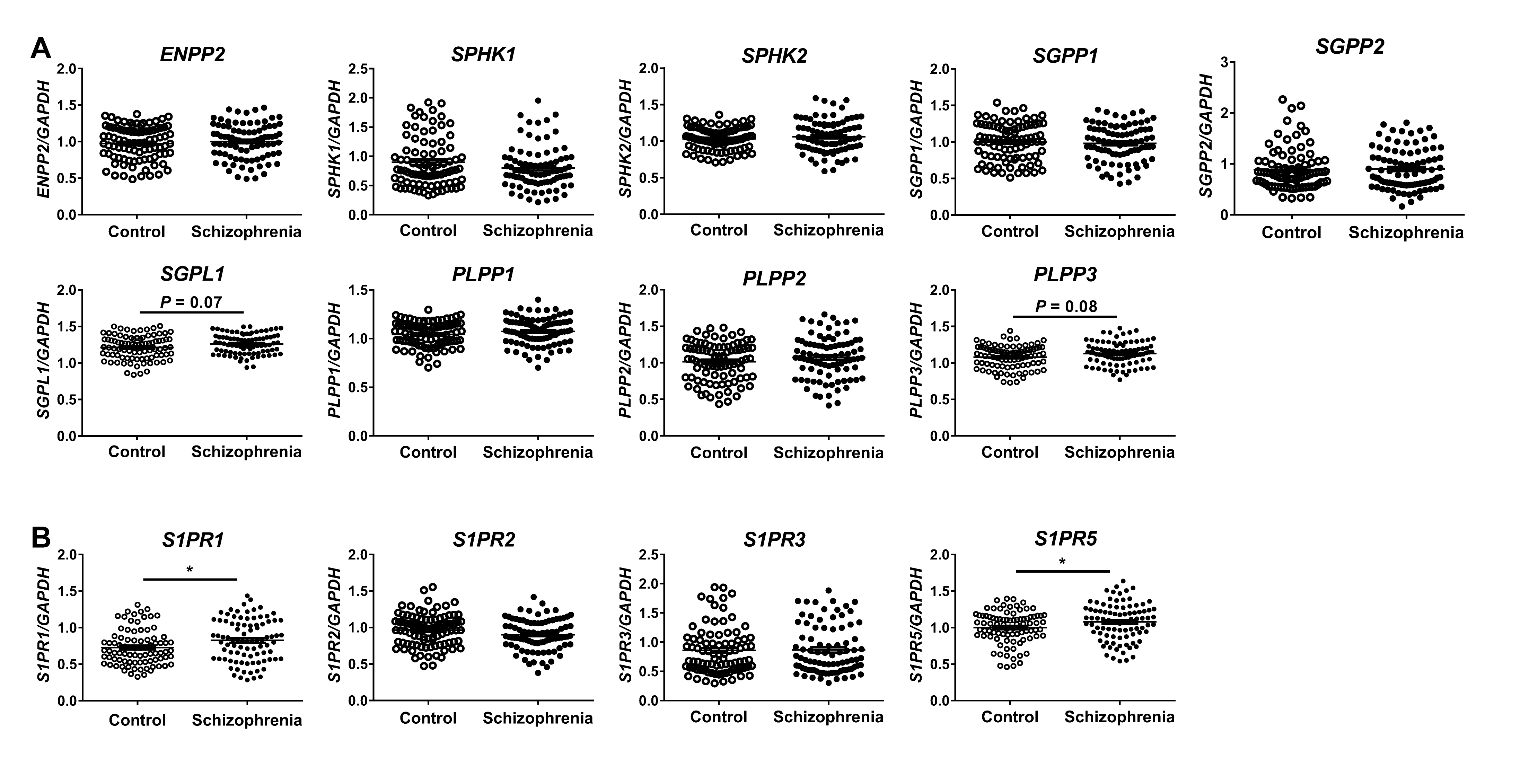


**Supplementary Figure S7. Expression analysis of other S1P metabolism-related and S1P receptor genes in the corpus callosum of subjects with schizophrenia**. The transcript expression levels of genes for (A) S1P-metabolic enzymes and (B) S1P receptors were normalized with *GAPDH* in the corpus callosum of subjects with schizophrenia (*n* = 91) and controls (*n* = 90). Data are represented as the mean ± SEM. **P* < 0.05, ***P* < 0.01. Differences between two groups were analyzed by Mann-Whitney U test.
